# Effects of combining cold exposure and compression on muscle recovery: a randomized crossover study

**DOI:** 10.3389/fphys.2025.1598075

**Published:** 2025-06-12

**Authors:** G. Millour, R. Lepers, A. Coste, C. Hausswirth

**Affiliations:** ^1^ beScored Institute, Sophia-Antipolis, France; ^2^ Laboratoire Motricité, Interactions, Performance, MIP, UR 4334, Nantes Université, Nantes, France; ^3^ Université Bourgogne Europe, Inserm, CAPS UMR 1093, Dijon, France

**Keywords:** cryotherapy, intermittent compression, inflammation, muscle fatigue, sports performance

## Abstract

**Introduction:**

The study aimed to evaluate the effects of combining lower-limb cold exposure and intermittent compression on optimizing post-exercise recovery.

**Methods:**

Fifteen male recreational athletes were recruited for a randomized crossover study comparing two recovery strategies: cryocompression and passive recovery, both applied in a supine position for 30 min. These interventions followed a high-intensity plyometric exercise and were repeated over the subsequent 2 days. Performance metrics included counter movement jumps and squat jumps, 30-s Wingate cycling test, maximal voluntary contraction (MVC) force of knee extensors, prolonged low-frequency force depression (PLFFD), inflammatory markers, and subjective assessments of muscle soreness and heaviness. Measurements were taken at four time points: pre-exercise, immediately post-recovery, 24 h post, and 48 h post.

**Results:**

Cryocompression significantly accelerated muscle recovery by reducing PLFFD and inflammation markers (salivary interleukin-1 beta and thigh circumference), while enhancing performance during MVC. Furthermore, perceived lower-limb heaviness, muscle soreness, and body pain decreased more rapidly with cryocompression at 24- and 48-h post-recovery. However, no significant differences were observed between the recovery strategies in cycling or jumping performance.

**Discussion:**

These findings underscore cryocompression as a promising recovery strategy for athletes seeking to mitigate exercise-induced muscle damage and restore performance. Further research is warranted to investigate the applicability of these results across diverse athletic populations.

## 1 Introduction

Physical exercise is widely recognized for its health benefits, playing a key role in preventing chronic diseases such as cardiovascular disorders, type 2 diabetes, and certain cancers. According to Khan et al. (2012), regular physical activity can reduce all-cause mortality by 20%–40%. However, intense or unfamiliar efforts can also lead to adverse effects such as muscle soreness (Wolska et al., 2023), sleep disturbances (Silva et al., 2019), or overall physical fatigue (Berger et al., 2024). These effects can impair performance and compromise the overall wellbeing of athletes. To counteract these negative outcomes, various recovery strategies have been developed, including nutritional supplementation (Gauche et al., 2006), post-exercise massages (Szabo et al., 2022), compression garments (Jakeman et al., 2010), whole-body cryotherapy (Pournot et al., 2011), far-infrared exposure (Hausswirth et al., 2011), and electrical stimulation (Babault et al., 2011).

Cold-based therapies, including cold-water immersion (CWI), cooling vests, and whole-body cryotherapy, are among the most used recovery methods, demonstrating significant effectiveness in alleviating muscle damage symptoms, including pain, stiffness, tenderness, and prolonged strength loss (Murray and Cardinale, 2015). Research suggests that CWI helps reduce post-exercise muscle damage, such as muscle fiber microtrauma, which is particularly prevalent after intense eccentric activities that place considerable strain on the muscles (Bleakley et al., 2012; Siqueira et al., 2018). When the body is exposed to cold, several biological mechanisms come into play. Cold exposure induces vasoconstriction, which reduces vascular permeability, limiting inflammatory processes and, consequently, alleviating muscle soreness (Bailey et al., 2007). Additionally, cryotherapy may enhance lactate clearance following high-intensity exercise, thereby reducing muscle fatigue (Bastos et al., 2012). Cryogenic temperatures may also have an analgesic effect, attributed to the activation of the endocrine system, which stimulates the release of endorphins while decreasing histamine and lactate concentrations in inflamed tissues (Wolska et al., 2023). These cold-induced benefits are mediated by several mechanisms: (1) Cryotherapy promotes the release of anti-inflammatory cytokines and growth factors that facilitate tissue repair; (2) Low temperatures influence the equilibrium between pro-oxidants and antioxidants, limiting cellular damage; (3) Cold stabilizes lysosomal membranes, inhibiting the release of active enzymes that may contribute to inflammation and muscle damage (Rose et al., 2017).

To optimize recovery, athletes may benefit from combining cryotherapy with compression of exercised limbs (Martínez-Guardado et al., 2020). Intermittent compression improves lymphatic and blood circulation, facilitating the removal of metabolites associated with muscle damage. Research has shown this approach to be effective in reducing perceived muscle fatigue, particularly after endurance events like ultramarathons (Hoffman et al., 2016). By applying pressure to dilated veins, compression reduces venous reflux, aiding blood return to the heart (Sarin et al., 1992). This method also activates the “muscle pump,” accelerating blood flow and delivering essential nutrients to recovering muscles (O'Riordan et al., 2023). Compression garments have also proven effective in enhancing recovery of force and power after exercise while minimizing muscle damage (Kraemer et al., 2001a). Furthermore, compression significantly impacts creatine kinase levels, a marker of muscle damage, supports tissue repair processes, and decreases oedema (Born et al., 2013; Kraemer et al., 2001b).

Based on these findings, this study aimed to assess the effectiveness of combining cold exposure and intermittent compression of the lower limbs in enhancing recovery following a plyometric muscle-damaging exercise in recreational athletes. The primary endpoint was the restoration of isometric muscle force, while secondary endpoints included changes in inflammatory markers, prolonged low-frequency force depression (PLFFD), physical performance (jump and anaerobic cycling tests), and subjective variables (delayed onset muscle soreness [DOMS], body pain, and heavy legs). We hypothesized that cryocompression would accelerate recovery kinetics and promote overall improvements in neuromuscular function and subjective recovery compared to passive recovery.

## 2 Materials and methods

### 2.1 Participants

The number of participants required for this study to address our hypotheses was calculated using a power analysis conducted with G*Power software (version 3.1.9.6). The sample size was determined based on data from a previous study comparing the effects of whole-body cryotherapy and passive recovery following a trail-running exercise that induced muscle damage (Hausswirth et al., 2011). Maximal isometric knee extensor force, assessed before and immediately after the exercise, as well as 24- and 48-h post-exercise, was used as the dependent variable for the power analysis. With an alpha risk of 5% (*α* = 0.05), a statistical power of 80% (1 – β = 0.80), and an intra-participant correlation coefficient of 0.6 between repeated measures, the required sample size was calculated to be 12 participants. To account for potential dropouts, 15 male recreational athletes were recruited for the study (mean ± standard deviation: age 39 ± 9 years; weight 77.6 ± 7.6 kg; height 1.80 ± 0.05 m; BMI 24.0 ± 2.2 kg/m^2^).

Inclusion criteria:

- Male participants aged 18–50.
- Participants in good health, i.e., free of musculoskeletal, cardiovascular, respiratory, or neurological disease or any other condition that may affect their ability to perform the required exercises.
- Participants must engage in regular physical activity, defined as 2–4 sessions per week of moderate to vigorous exercise.

Exclusion criteria:

- Participants with any contraindications for the use of the CryoPush® device (CRYO-NOV, Saulx-les-Chartreux, France), as outlined by the manufacturer.
- Participants who have taken anti-inflammatory medications, corticosteroids, or any other drugs that could potentially influence muscle recovery or inflammation within 2 weeks prior to the study.

All participants were fully informed about the study’s risks and benefits and provided written informed consent. The study protocol was approved by the National Ethics Committee (CER STAPS) in compliance with the Declaration of Helsinki (ethical approval: IRB00012476-2024-23-05-314).

### 2.2 Study design

**FIGURE 1:**
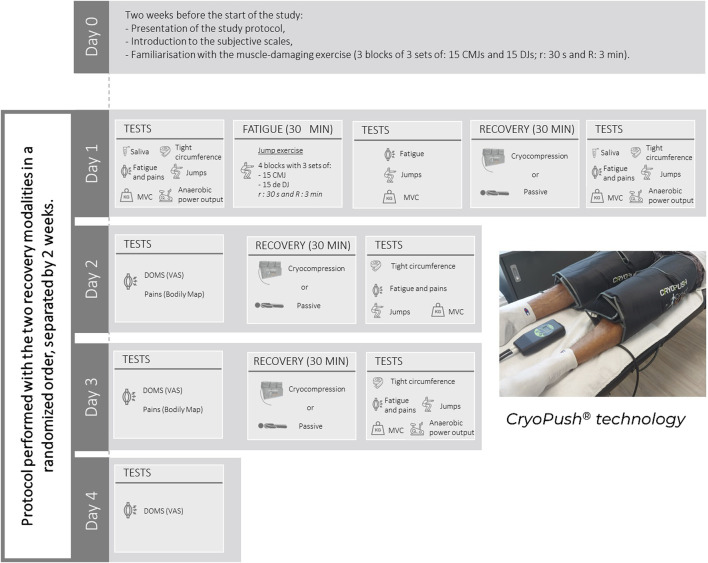
Schematic representation of the experimental protocol and illustration of the CryoPush® technology. MVC, Maximal Voluntary Contractions; CMJ, Counter Movement Jumps; DJ, Drop Jumps; DOMS, Delayed Onset of Muscle Soreness; r, Recovery time between sets; R, Recovery time between blocks; VAS, Visual Analog Scale.

We conducted a randomized, controlled, crossover study. The study was conducted at the beScored Institute (Valbonne, France), and the recruitment period lasted from May to June 2024. The experiment itself took place between June and September 2024. Each participant experienced two recovery modalities after plyometric muscle-damaging exercise in a random order: recovery using the CryoPush® device (V3) (Figure 1) or passive recovery, both in a supine position for 30 min. During cryocompression sessions, two sleeves were positioned on the participants’ lower limbs, covering the upper thigh to mid-calf area. Pre-cooled, reusable cold packs, stored at a standardized freezer temperature (−18°C), were inserted into the sleeves connected to a pump. This pump delivered intermittent compression, alternately applied to each leg, progressively reaching a pressure of 60 mmHg. This setup was designed to deliver both cooling and compression effects simultaneously. Fatigue and recovery sessions were preceded and followed by a standardized battery of tests, which will be detailed in the *Materials and Measurements* section (Figure 1). Evaluation days were distributed across 2 weeks (Phase 1 and Phase 2), differentiated by the assigned recovery modality. A 2-week washout period was implemented between phases to ensure complete recovery. To control for confounding factors related to additional fatigue, participants were instructed to avoid any physical training during the 2 days preceding the start of Phase 1 and Phase 2 and during the 2 days following the muscle-damaging exercise. A familiarization session with the muscle-damaging protocol was conducted 2 weeks before the study to habituate participants to the movements and produce a repeated bout effect, reducing muscle disruptions led by unaccustomed eccentric exercise.

The muscle-damaging exercise, conducted only on the first evaluation day of Phase 1 and Phase 2, consisted of repeated jump exercises, specifically counter movement jumps (CMJ) and drop jumps (DJ). During the CMJ, participants started from a standing position and initiated a downward movement until their knees were flexed at a 90° angle. This flexion was immediately followed by an extension of the lower limbs, leading to a propulsion phase to reach maximum jump height. For the DJ, participants started on a platform 30 cm high. They were instructed to leave the platform, landing with full-foot contact on the ground and flexing their knees to absorb the impact. Immediately after landing, they had to jump vertically, aiming to reach maximum height as quickly as possible. These plyometric exercises are known to induce significant dynamic and localized muscle fatigue (Skurvydas et al., 2002; Fowler et al., 1997). Participants performed four blocks of three sets, each consisting of 15 CMJ and 15 DJ, for a total of 90 jumps per block and 360 jumps overall. Recovery between sets was 30 s, and recovery between blocks was 3 min. During the familiarization session conducted 2 weeks before the study, participants completed three blocks of three sets of 15 CMJ and 15 DJ, totaling 270 jumps.

Recovery sessions were conducted after the muscle-damaging exercise and the following 2 days due to the delayed onset of muscle soreness (DOMS). As previously mentioned, recovery involved either treatment with CryoPush® technology or passive recovery. Regardless of the recovery modality, each session lasted 30 min.

### 2.3 Materials and measurements

#### 2.3.1 Inflammatory markers

To assess inflammatory responses, salivary concentrations of the pro-inflammatory cytokine interleukin-1 beta (IL-1β) were measured. Samples were collected using Salivettes (Sarstedt AG & Co. KG, Nümbrecht, Germany) at two time points during each phase: before exercise (Day 1) and 1 h post-exercise. This timing was based on prior evidence suggesting that salivary cytokine levels typically peak 45–100 min after a physiological stressor (Szabo and Slavish, 2021). Participants were instructed to chew on a cotton swab for 3 min to stimulate saliva production. Samples were immediately frozen at −20°C and later analyzed by a certified biomedical laboratory (Cerballiance, Grand Saint-Jean, Cagnes-sur-Mer, France). Each tube was labeled with the date, time, and participant ID for traceability. After completion of the study, all samples were thawed and centrifuged at 3,000 rpm for 10 min at room temperature, following a standardized protocol. IL-1β concentrations were then analyzed using a high-sensitivity enzyme-linked immunosorbent assay (ELISA), a standard immunoassay technique that quantifies target proteins through antigen–antibody interactions. Results were reported by the laboratory’s medical biologist. All remaining biological material was eliminated via the regulated medical waste (DASRI) stream and incinerated to prevent any contamination risk.

In addition to this biological marker, anthropometric measurements of the right thigh circumference were taken using a tape measure according to a standardized procedure at the beginning of Day 1 and after the recovery periods on Days 1, 2, and 3. These measurements can serve as indicators of acute changes in thigh volume (i.e., oedema) that may occur following eccentric exercises (Fielding et al., 2000). All measurements were conducted by the same operator. First, the operator marked the area on the thigh where the circumference was maximal. To ensure consistency across blocks, the distance between the top of the patella and the maximal circumference was measured and replicated during both testing phases for each participant.

#### 2.3.2 Prolonged low-frequency force depression

Following the anthropometric measurements, PLFFD was assessed using the scientifically validated Myocene® device (Myo1, Myocene, Liège, Belgium; Ridard et al., 2022). Measurements were taken before and after the muscle-damaging exercise, and after the recovery periods on Days 1, 2, and 3. Participants were seated on the device with their tibia in contact with the Myo-sensor, a force sensor that records forces at a frequency of 4 kHz. Three surface electrodes (MyoPro-1, Myocene, Liège, Belgium) were used for neuromuscular electrical stimulation (NMES). The cathode (5 × 10 cm) was placed transversely over the proximal third of the rectus femoris, and the two anodes (5 × 5 cm) were positioned longitudinally over the motor points of the vastus lateralis and vastus medialis, respectively, as identified by anatomical landmarks and palpation, in accordance with the recommendations of Botter et al. (2011). The NMES protocol delivered biphasic rectangular (square wave) pulses with a constant pulse width of 400 μs, as pre-programmed in the Myocene® system. These parameters, including pulse duration, frequency, and current intensity, were fixed and non-adjustable by the user, and were identical for all participants. Each stimulation series consisted of three successive stimulations: (1) a single twitch, (2) low-frequency tetanic stimulation (5 pulses at 20 Hz), and (3) high-frequency tetanic stimulation (18 pulses at 120 Hz). A 1-s interval separated each stimulation. A total of 16 stimulation series were administered, spaced 5 s apart, with the stimulation intensity progressively increased in 1 mA steps per series (from 25 mA to 40 mA). The total duration of the PLFFD assessment with the device was 2 min. The Myocene® software integrates a specific algorithm that automatically analyzes the force signals during each stimulation series. Within each series, the peak force from the low-frequency stimulation was divided by the peak force from the high-frequency stimulation, generating a ratio that reflects the extent of low-frequency fatigue. The Powerdex, expressed as a percentage, corresponds to the median value of these 16 low-/high-frequency force ratios and serves as the outcome measure. A lower Powerdex indicates a greater degree of PLFFD.

#### 2.3.3 Explosive performance during jumps

After a 5-min cycling warm-up with free power and cadence on a cycle ergometer (LC6 Novo, Monark Exercise AB, Vansbro, Sweden), which was previously adjusted to each participant’s morphology, we assessed participants’ jump performance. For this, they were required to perform three repetitions of squat jumps (SJ) and three repetitions of CMJ, with 30 s of rest between each repetition. During the SJ, the participant flexed their knees to 90° and held this position for 3 s with their hands on their hips. Then, they had to jump as high as possible without performing any counter movement (i.e., downward impulse). The flight time was recorded using K-Delta force platforms (Kinvent®, Montpellier, France) connected to the Kinvent® application (Bagchi et al., 2024). Based on flight time, the jump height was calculated (Albano et al., 2019). These jump tests were repeated after the muscle-damaging exercise and following each recovery period on Days 1, 2, and 3.

#### 2.3.4 Maximum isometric force of the knee extensor muscles

Five minutes after each jump exercise described previously, the maximal isometric muscle force of the right knee extensors was evaluated during maximal voluntary contractions (MVC, in N). The tests were performed on a leg extension bench equipped with a steel link chain and an S-force sensor (ME-Meßsysteme GmbH, Germany, model KD40s, ±5 kN). The chain length was adjusted so that the knee angle was approximately 90° for each participant. The S-force sensor was connected to a computer using a digital measurement amplifier (GSV-3USB) and data acquisition software (GSVmulti, version 1.39.6.8). This software enabled real-time visualization and recording of the data sampled at 100 Hz. Two tests were conducted: (i) three maximal 3-s trials to evaluate the maximal voluntary isometric contraction and (ii) a 30-s trial with maximal involvement to assess average muscle performance. Each trial was separated by a 45-s rest period to allow recovery.

#### 2.3.5 Anaerobic power output

Finally, a Wingate test was performed on the cycle ergometer to measure anaerobic performance at the start and end of Day 1 and at the end of Day 3 (each time 5 min after the MVC). The seat and handlebar settings were recorded after the warm-up and reproduced for each trial to ensure proper reproducibility of positioning between tests. Participants were required to remain seated on the saddle throughout the entire Wingate test to ensure consistent body positioning. The cycle ergometer was connected to a computer, and the Monark software was used to design the Wingate protocol, control the bike, and record the data sampled at 1 Hz. After 3 min of cycling at free power and cadence, the Wingate test began with an initial 15-s period against a resistance of 10 N, followed directly by a maximal 30-s effort against a resistance dependent on the participant’s body weight (7.5% of body weight). Participants were encouraged to maintain the highest possible pedaling cadence for 30 s. At the end of the test, peak power output and average power output were calculated.

#### 2.3.6 Subjective variables

Participants responded to a visual analog scale (VAS) regarding their sensation of heavy legs following the jump tests (Schaefer et al., 2003). The front of the scale displayed the question, such as “How do your legs feel at this moment?” and a linear gauge with two marks at the extremes (at the bottom: “very, very light”; at the top: “very, very heavy”) allowed participants to assess their leg heaviness by moving the indicator along the scale. On the reverse side, a numerical scale ranging from 0 (representing “very, very light”) to 10 (“very, very heavy”) enabled the investigator to read the value corresponding to the perceived leg heaviness without the participant being aware of it. Additionally, the days following the muscle-damaging exercise (Day 2, Day 3, and Day 4), participants indicated their level of soreness (DOMS; Mattacola et al., 1997) using a similar VAS. Finally, the overall pain intensity was evaluated using a digital body map programmed using MATLAB with the Psychtoolbox extension at the beginning and immediately after the recovery periods on Days 1, 2, and 3 (Rigoard et al., 2021). Participants used the mouse to place colored circles over the painful areas on a body silhouette. They could choose from three circle sizes (small, medium, and large) depending on the extent of the pain. Participants also had the option to select the front or back of the body and could erase and redo their markings if needed. A color code was used to represent pain intensity: red for severe pain, dark pink for moderate pain, and light pink for mild pain. The pain intensity was assigned the following coefficients: one for mild pain, two for moderate pain, and three for severe pain. Similarly, the size of the circle was associated with coefficients: one for a small area, two for a medium area, and three for a large area. The pain score for each area was calculated by multiplying the area by the intensity:

Pain score for each area =Area×Intensity

The global pain score was obtained by summing the scores for all painful areas:

$$Global\;pain\;score=\sum_{i=1}^{n}\left({Area}_{i}\times {Intensity}_{i}\right)\;$$

where *n* represents the total number of painful areas indicated by the participant.

### 2.4 Statistical analysis

To analyze the quantitative data, a two-way repeated-measures ANOVA (recovery modality × time) was performed to compare the effects of the recovery modality (Cryocompression vs. Passive) across the different time points (e.g., Pre, Post, Post 24 h, and Post 48 h). The normality of the distribution, homogeneity of variances, and the sphericity assumption were tested using Shapiro-Wilk, Levene, and Mauchly’s tests, respectively. When the sphericity assumption was violated, the Greenhouse-Geisser correction was applied. In the event of a significant recovery modality × time interaction in the ANOVA, Tukey’s HSD *post hoc* tests were performed to identify pairwise differences. The measurements taken between the muscle-damaging and recovery protocols ensured that the level of fatigue induced by the exercise was consistent across both phases, confirming that participants exerted a similar effort in each session. Paired Student's t-tests or Wilcoxon tests in cases of non-homogeneity of the variances or non-normality of the distribution were used to compare changes in muscle force, jump performance, and PLFFD (Myocene®) before and after the muscle-damaging exercise in the two experimental conditions (Cryocompression vs. Passive). The level of significance was set at p < 0.05.

## 3 Results

### 3.1 Muscle-damaging exercise: performance and fatigue induced by exercise

The average jump height during the muscle-damaging test was not statistically different for the two experimental conditions (Passive: 17.5 ± 4.8 cm vs. Cryocompression: 18.1 ± 3.9 cm, p = 0.26). After the muscle-damaging exercise, muscle force during the 3-s MVC was reduced by 20% ± 12% and 20% ± 10% for the Passive and Cryocompression conditions, respectively (*p* = 0.91). During the 30-s MVC, force decreased by 27% ± 8% and 21% ± 10% for the Passive and Cryocompression conditions, respectively (*p* = 0.08). The average jump height decreased by 5% ± 7% for SJ and 4% ± 7% for CMJ for the Passive condition and by 8% ± 5% for SJ and 8% ± 4% for CMJ for the Cryocompression condition (*p* = 0.32 for SJ and *p* = 0.10 for CMJ). The muscle fatigue index, highlighted by the reduction in Powerdex, showed a decrease of 31% ± 9% for the Passive condition and 29% ± 15% for the Cryocompression condition (*p* = 0.65).

### 3.2 Inflammatory markers

The IL-1β levels, collected before exercise and 1 h after the muscle-damaging protocol, revealed a significant effect of the recovery modality (*p* = 0.008), a period effect (*p* < 0.001), and an interaction effect (*p* = 0.007) (Table 1). *Post-hoc* tests highlighted a significant increase in IL-1β levels after the session compared to pre-session values in both the Passive (+6.9%, *p* < 0.001) and the Cryocompression conditions (+5.5%, *p* < 0.001). Additionally, a significant difference was observed between the post-session values of the two phases (p = 0.033).

**TABLE 1:** Mean (± standard deviation) of IL-1β levels (pre and post session 1) and thigh circumference (pre, post, 24 h post, and 48 h post) during the Passive and Cryocompression conditions.

Variables	Condition	Pre	Post	Post 24 h	Post 48 h	Modality	Period	Interaction
IL-1β level (pg/mL)	Passive	0.51 ± 0.10	4.02 ± 1.55***	-	-	0.008	<0.001	0.007
	Cryocompression	0.50 ± 0.10	3.25 ± 1.45***^,$^	-	-			
Thigh circumference (cm)	Passive	57.7 ± 4.0	58.0 ± 4.1*	57.9 ± 4.0	57.9 ± 3.9	0.016	0.88	<0.001
	Cryocompression	57.7 ± 4.0	57.4 ± 4.2^$^	57.6 ± 4.1	57.6 ± 4.1			

The statistical significance of the recovery modality factor, period factor, and interaction effect are indicated (significant effects and pairwise differences are shown in bold). *different from pre-session values (p < 0.05); ***different from pre-session values (p < 0.001); ^$^different from post-session Passive values (p < 0.05).

Thigh circumference measurements, used to assess muscle oedema before and after recovery as well as at 24- and 48-h post-recovery, revealed a significant recovery modality effect (p = 0.016) and an interaction effect (p < 0.001) (Table 1). *Post-hoc* analyses showed a decrease in thigh circumference following recovery for the Cryocompression condition (*p* = 0.044). In contrast, a slight, non-significant increase was observed for the Passive condition. This led to a significant difference in post-recovery values between the two conditions (*p* = 0.048).

### 3.3 Prolonged low-frequency force depression

**FIGURE 2:**
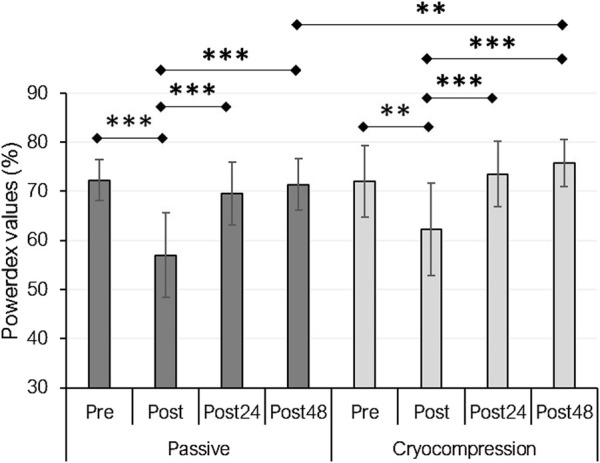
Mean (± standard deviation) of Powerdex values measured with the Myocene® device at pre, post, post-24 h, and post-48 h for the Passive and Cryocompression conditions. **p < 0.01, ***p < 0.001.

PLFFD, assessed using Powerdex values from the Myocene® device, showed a recovery modality effect (*p* = 0.014), a period effect (*p* < 0.001), and an interaction effect (*p* = 0.034) (Figure 2). For the Passive condition, Powerdex decreased immediately post-session one compared to pre-session values (−21%, *p* < 0.001) but subsequently increased post-24 h (+22%, *p* < 0.001) and post-48 h (+25%, *p* < 0.001). A similar trend was observed in the Cryocompression condition, with a smaller initial decline immediately post-session (−14%, *p* = 0.009), followed by an increase at post-24 h (+18%, *p* < 0.001) and post-48 h (+22%, *p* < 0.001). *Post-hoc* tests highlighted significantly higher Powerdex values at post-48 h for the Cryocompression than the Passive condition (+6.1%, *p* = 0.002).

### 3.4 Maximum force of the knee extensor muscles

**FIGURE 3:**
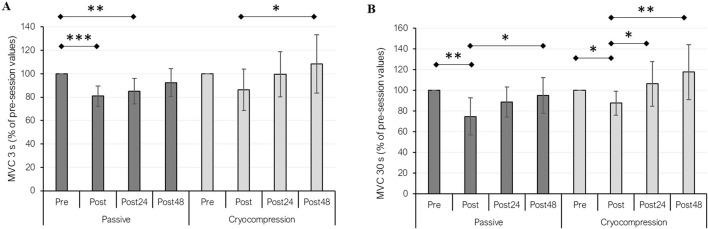
Mean (± standard deviation) of performance during 3-s **(A)** and 30-s **(B)** maximal voluntary contractions (MVC) at pre, post, post-24 h, and post-48 h for the Passive and Cryocompression conditions. *p < 0.05, **p < 0.01, ***p < 0.001.

MVC force performance was evaluated over 3 s (Figure 3A) and 30 s (Figure 3B). The data were normalized to absolute values, using the pre-session values of both conditions as the reference (set to 100%). For the 3-s MVC test, we identified a recovery modality effect (*p* = 0.030), a period effect (*p* < 0.001), and a significant interaction between factors (*p* = 0.014). Post-hoc analyses revealed a significant performance decrease in the Passive condition between pre and post (−19%, *p* < 0.001) and between pre- and post-24 h (−15%, *p* = 0.003). In contrast, no such decline was observed during the Cryocompression condition. Furthermore, a significant performance improvement between post and 48 h post was noted during the Cryocompression phase (+26%, *p* = 0.014). For the 30-s MVC test, statistical analyses also showed a recovery modality effect (*p* = 0.003), a period effect (*p* < 0.001), and a significant interaction (*p* = 0.005). In the Passive condition, performance decreased between pre and post (−25%, *p* = 0.003) but improved significantly between post and post-48 h (+27%, *p* = 0.012). Similarly, for the Cryocompression condition, performance declined between pre and post (−12%, *p* = 0.028), but significant improvements were observed at post-24 h (+21%, *p* = 0.023) and post-48 h (+34%, *p* = 0.001) compared to post-session values.

### 3.5 Explosive performance during jumps

Performance during jumps was evaluated based on the average and maximum height measured during the SJ and CMJ (Table 2). Despite a period effect (*p* < 0.001), no significant interaction was found for these variables.

**TABLE 2:** Mean (± standard deviation) of the average and maximum height during the three jumps performed at pre, post, post-24 h, and post-48 h during the Passive and Cryocompression conditions.

Variables	Condition	Pre	Post	Post 24 h	Post 48 h	Modality	Period	Interaction
Average SJ height (cm)	Passive	26.5 ± 3.7	24.0 ± 2.9	26.0 ± 3.7	26.6 ± 4.5	0.270	**< 0.001**	0.249
	Cryocompression	27.5 ± 4.1	23.8 ± 4.1	26.3 ± 3.4	27.6 ± 3.1			
Maximal SJ height (cm)	Passive	27.8 ± 4.0	25.4 ± 3.5	27.2 ± 3.7	27.8 ± 4.6	0.434	**< 0.001**	0.109
	Cryocompression	28.9 ± 4.4	24.9 ± 4.1	27.1 ± 3.2	28.8 ± 3.3			
Maximal CMJ height (cm)	Passive	30.1 ± 4.5	27.8 ± 4.0	29.9 ± 4.1	30.3 ± 4.5	0.775	**< 0.001**	0.331
	Cryocompression	30.8 ± 4.4	27.1 ± 4.0	29.9 ± 3.9	30.9 ± 3.9			
Average CMJ height (cm)	Passive	31.1 ± 4.3	28.6 ± 4.3	30.8 ± 3.9	31.5 ± 4.7	0.827	**< 0.001**	0.484
	Cryocompression	31.6 ± 4.3	28.0 ± 4.4	30.7 ± 4.1	32.1 ± 4.3			

The statistical significance of the recovery modality factor, period factor, and interaction effect are indicated (significant effects are shown in bold).

SJ, squat jump; CMJ, counter movement jump.

### 3.6 Anaerobic power output

The analysis of average and maximal power during the Wingate test revealed a significant period effect in both experimental phases. However, there were no recovery modality and interaction effects (Table 3).

**TABLE 3:** Mean (± standard deviation) of average and maximal power output during the Wingate test conducted at pre, post and post-48 h for the Passive and Cryocompression conditions.

Variables	Condition	Pre	Post	Post 48 h	Modality	Period	Interaction
Average power (W)	Passive	615 ± 78	583 ± 79	604 ± 75	0.941	**< 0.001**	0.069
	Cryocompression	607 ± 70	574 ± 76	614 ± 67			
Maximal power (W)	Passive	783 ± 118	769 ±126	812 ± 138	0.958	**< 0.001**	0.156
	Cryocompression	805 ± 138	754 ± 132	797 ± 111			

The statistical significance of the recovery modality factor, period factor, and interaction effect are indicated (significant effects are shown in bold).

### 3.7 Subjective variables

**FIGURE 4:**
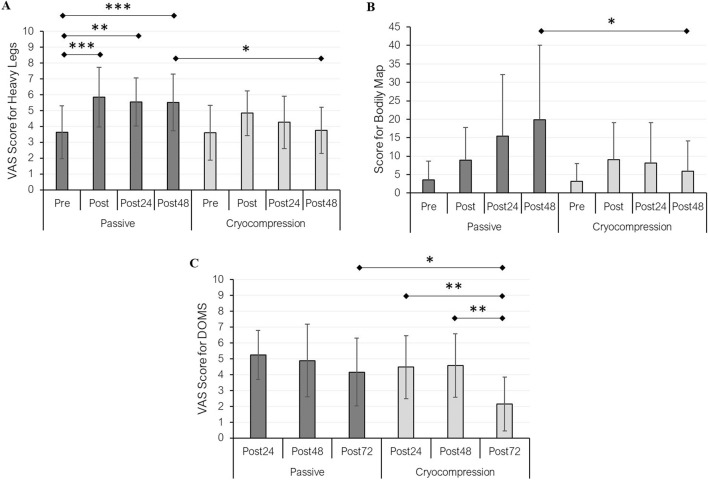
Mean (± standard deviation) of heavy legs **(A)** and pain **(B)** perceptions at pre, post, post-24 h, and post-48 h for the passive and cryocompression conditions. DOMS (delayed onset muscle soreness) was assessed at post-24 h, post-48 h, and post-72 h during both experimental conditions **(C)**. *p < 0.05, **p < 0.01, ***p < 0.001.

Subjective variables were used to assess the perception of heavy legs (Figure 4A), body pain (Figure 4B), and DOMS (Figure 4C) during both experimental conditions. Regarding the perception of heavy legs, a recovery modality effect (*p* = 0.005), a period effect (*p* < 0.001), and an interaction effect (*p* = 0.019) were observed. Pairwise comparisons revealed a significant difference between pre- and post-session conditions (+61%, *p* < 0.001), as well as between pre and post 24-h (+53%, *p* = 0.001) and post-48 h (+52%, *p* < 0.001) in the Passive condition. No significant differences were found in the Cryocompression condition. A significant difference between post-48 h measurements in the two conditions was also found (*p* = 0.012), with legs feeling lighter after using the Cryocompression system. The analysis of body pain showed a recovery modality effect (*p* = 0.007), a period effect (*p* = 0.028), and an interaction effect (*p* = 0.007). *Post-hoc* tests revealed significantly lower pain levels 48 h after the muscle-damaging exercise when using the cryocompression system (*p* = 0.046). In addition, for DOMS, we found significant effects for recovery modality (*p* < 0.001), period (*p* < 0.001), and interaction (*p* = 0.049). Post-hoc tests identified a reduction in DOMS at post-72 h compared to post-24 h (−52%, *p* = 0.008) and post-48 h (−53%, *p* = 0.002) exclusively during the Cryocompression condition. A significant difference between the two post 72-h conditions was also found (*p* = 0.018), with less soreness following cryocompression. Pearson’s correlation analyses revealed that body pain was significantly associated with several variables, mostly in the Passive condition. A positive correlation was found with thigh circumference at the end of Day 1 (r = 0.60, p = 0.031) and with leg heaviness after recovery on Days 1, 2, and 3 (r = 0.56–0.71, all p < 0.05). Body pain before recovery was also strongly correlated with DOMS at post-48 h (r = 0.73, p = 0.005). In the Cryocompression condition, body pain was significantly correlated with DOMS at post-24 h (r = 0.76, p = 0.002) and post-48 h (r = 0.69, p = 0.008). No significant correlations were observed with IL-1β in either condition.

## 4 Discussion

The present study aimed to evaluate the effects of recovery using a cryocompression technology compared to passive recovery, after a muscle-damaging exercise. We found that cryocompression accelerated recovery by reducing muscle inflammation, PLFFD, soreness, and pain, while improving the knee extensors’ maximal force. However, no significant differences were observed between the two experimental conditions during the jumping and Wingate cycling performance tests.

The study demonstrated that the muscle-damaging exercise reduced quadriceps force and jump height while increasing PLFFD. Such stressful activities, including plyometric exercises, elevate cytosolic calcium levels, increase sarcolemma permeability, cause muscle fiber oedema, disrupt cellular structures, and result in soreness and reduced force-generating capacity (White and Wells, 2013). The observed 20%–30% decline in force during MVC after the demanding exercise falls within the intermediate range of values reported in previous studies after a trail running race (Gauche et al., 2006; Hausswirth et al., 2011), confirming the effectiveness of the current protocol in inducing muscle fatigue.

Following the muscle-damaging exercise, cryocompression significantly reduced IL-1β levels and thigh circumference, a marker of oedema, suggesting that the combined effects of cold exposure and compression enhance the management of inflammatory responses. The reduction in extracellular fluid accumulation aligns with findings from previous studies on cold exposure (Eston and Peters, 1999; White and Wells, 2013) and compression (Kraemer et al., 2001a; Kraemer et al., 2001b; Born et al., 2013). The observed effects could be attributed to vasoconstriction, which may reduce vascular permeability and limit inflammatory processes (Bailey et al., 2007; Born et al., 2013), potentially amplified by the synergy of both recovery techniques. Similarly, Pournot et al. (2011) observed a decrease in IL-1β, a classical marker of exercise-induced muscle damage, when athletes used whole-body cryotherapy compared to passive recovery after a simulated trail running race. Furthermore, compression garments have been shown to attenuate creatine kinase and myoglobin elevations following eccentric exercise (Mizuno et al., 2017; Kraemer et al., 2001a; Kraemer et al., 2001b). However, previous studies reported no significant effect of compression on pro-inflammatory markers such as TNF-α, IL-1β, and IL-6 (Pruscino et al., 2013; Kim et al., 2017), suggesting that the reduction in IL-1β observed with cryocompression is primarily driven by cold exposure.

The muscle-damaging exercise increased PLFFD, likely reflecting excitation–contraction coupling failure due to reduced Ca^2+^ release within muscle fibers, consistent with the findings of Ridard et al. (2022). However, it is important to note that the Myocene® device estimates PLFFD using brief trains of low- and high-frequency stimuli, rather than sustained tetanic contractions. These short-duration trains may not induce full Ca^2+^ saturation and could therefore underestimate both the magnitude and duration of PLFFD. As shown by Ruggiero et al. (2019), protocols using very short trains (such as doublets) lead to a faster recovery of the low-frequency fatigue ratio (within 48 h), whereas longer tetanic stimulations reveal persistent impairments up to 96 h following eccentric exercise. This methodological limitation should be considered when interpreting the observed PLFFD values, as the actual level of neuromuscular fatigue may be more pronounced. Nonetheless, PLFFD was significantly reduced 48 h post-exercise when recovery was facilitated with cryocompression compared to passive recovery, suggesting a beneficial effect on muscle fatigue.

Moreover, the force of knee extensors during MVC was impaired on the first day after passive recovery, while recovery kinetics was accelerated with cryocompression at both 24 h and 48 h post-exercise. According to Carroll et al. (2017), a reduction in maximal force during an isometric MVC is considered the clearest indicator of performance fatigability, which is consistent with the lower muscle fatigue at post-48 h for the Cryocompression condition highlighted by the Myocene® results. This reduced fatigability is likely attributed to the combined effects of cold exposure and compression, which may alleviate muscle damage and promote tissue repair (Born et al., 2013; Rose et al., 2017). Previous studies have also shown improved MVC performance following recovery with cryotherapy (Hausswirth et al., 2011) or compression (Kim et al., 2017) immediately after muscle-damaging exercise and in the subsequent days.

Despite these positive findings, certain variables did not differ significantly between recovery modalities, such as performance during the jump tests (SJ and CMJ) or the Wingate anaerobic cycling test. Pruscino et al. (2013) also reported no improvement in explosive performance during jumps when recovery was performed with compression garments. In addition, Vieira et al. (2015) observed no effect of whole-body cryotherapy on jump performance after high-intensity exercise, including concentric and eccentric efforts, while the impact of cold exposure on anaerobic power output is controversial (Crowe et al., 2007; Al-Nawaiseh et al., 2016). This could be attributed to the neuromuscular adaptations required for these types of efforts, which may not be directly influenced by inflammation or muscle fatigue but rather depend on central nervous system mechanisms. Nevertheless, preserving performance in these metrics, alongside enhanced MVC recovery, suggests that cryocompression provides a competitive advantage by maintaining muscle force capacity.

Finally, subjective assessments of pain and the sensation of heavy legs aligned with the objective data, further supporting the benefits of using cryocompression for recovery. The positive effects of cryotherapy (Burgess and Lambert, 2010; Wolska et al., 2023) and compression (Kraemer et al., 2001a; Kraemer et al., 2001b; Kim et al., 2017) on perceived soreness are well-documented in the literature, highlighting their role in improving recovery outcomes and enhancing athletes’ comfort during the post-exercise period. This combination of cold exposure and compression not only facilitates a faster return to training, but also could help minimize the risk of injury by reducing muscle soreness and promoting quicker recovery (Kraemer et al., 2001a). The observed correlations between pain levels, DOMS, leg heaviness, and thigh circumference highlight the utility of the body map, which can be considered a reliable tool for assessing muscle pain and accurately identifying its location.

The study does have some limitations. The exclusive inclusion of male participants limits the generalizability of the findings to other populations, such as female athletes or individuals from various sports disciplines and performance levels. Additionally, the study did not include a direct comparison with other established recovery methods, such as whole-body cryotherapy or active recovery, which could provide a clearer understanding of the relative effectiveness of cryocompression compared to other popular recovery strategies. Finally, the study only included short-term assessments of recovery, and further research is needed to evaluate the long-term effects of cryocompression on muscle function and overall training outcomes.

## 5 Conclusion

In conclusion, this study demonstrates that cryocompression is an effective intervention to accelerate recovery following muscle-damaging exercise. The results showed significant reductions in inflammatory markers, PLFFD, and muscle soreness, with improvements in maximal knee extensor force capacity. These findings support the hypothesis that cryocompression can enhance recovery by mitigating the physiological impacts of intense eccentric exercise. These positive effects suggest that cryocompression systems may serve as a valuable recovery tool for athletes engaging in high-intensity training. Future studies should explore the long-term effects and mechanisms underlying cryocompression’s benefits, as well as its applicability in various athletic populations.

## Data Availability

The original contributions presented in the study are included in the article/supplementary material, further inquiries can be directed to the corresponding author.

## References

[B1] Albano D. Coppola S. Vastola R. (2019). Vertical jump performance in Italian elite trials athletes. J. Phys. Educ. Sport 19, 2110–2114. 10.7752/jpes.2019.s6316

[B2] Al-Nawaiseh A. M. Pritchett R. C. Bishop P. A. (2016). Enhancing short-term recovery after high-intensity anaerobic exercise. J. Strength Cond. Res. 30, 320–325. 10.1519/JSC.0000000000001060 26815173

[B3] Babault N. Cometti C. Maffiuletti N. A. Deley G. (2011). Does electrical stimulation enhance post-exercise performance recovery? Eur. J. Appl. Physiol. 111, 2501–2507. 10.1007/s00421-011-2117-7 21847574

[B4] Bagchi A. Raizada S. Thapa R. K. Stefanica V. Ceylan H. İ. (2024). Reliability and accuracy of portable devices for measuring countermovement jump height in physically active adults: a comparison of force platforms, contact mats, and video-based software. Life 14 (11), 1394. 10.3390/life14111394 39598192 PMC11595741

[B5] Bailey D. M. Erith S. J. Griffin P. J. Dowson A. Brewer D. S. Gant N. (2007). Influence of cold-water immersion on indices of muscle damage following prolonged intermittent shuttle running. J. Sports Sci. 25, 1163–1170. 10.1080/02640410600982659 17654228

[B6] Bastos F. N. Vanderlei L. C. M. Nakamura F. Y. Bertollo M. Godoy M. F. Hoshi R. A. (2012). Effects of cold-water immersion and active recovery on post-exercise heart rate variability. Int. J. Sports Med. 33, 873–879. 10.1055/s-0032-1301905 22722961

[B7] Berger N. J. Best R. Best A. W. Lane A. M. Millet G. Y. Barwood M. (2024). Limits of ultra: towards an interdisciplinary understanding of ultra-endurance running performance. Sports Med. 54, 73–93. 10.1007/s40279-023-01936-8 37751076

[B8] Bleakley C. McDonough S. Gardner E. Baxter G. D. Hopkins J. T. Davison G. W. (2012). Cold‐water immersion (cryotherapy) for preventing and treating muscle soreness after exercise. Cochrane Database Syst. Rev. 2012 (2), CD008262. 10.1002/14651858.CD008262.pub2 22336838 PMC6492480

[B9] Born D. P. Sperlich B. Holmberg H. C. (2013). Bringing light into the dark: effects of compression clothing on performance and recovery. Int. J. Sports Physiol. Perform. 8, 4–18. 10.1123/ijspp.8.1.4 23302134

[B10] Botter A. Oprandi G. Lanfranco F. Allasia S. Maffiuletti N. A. Minetto M. A. (2011). Atlas of the muscle motor points for the lower limb: implications for electrical stimulation procedures and electrode positioning. Eur. J. Appl. Physiol. 111, 2461–2471. 10.1007/s00421-011-2093-y 21796408

[B11] Burgess T. L. Lambert M. I. (2010). The efficacy of cryotherapy on recovery following exercise-induced muscle damage: invited review article. Int. SportMed J. 11, 258–277.

[B12] Carroll T. J. Taylor J. L. Gandevia S. C. (2017). Recovery of central and peripheral neuromuscular fatigue after exercise. J. Appl. Physiol. 122, 1068–1076. 10.1152/japplphysiol.00775.2016 27932676

[B13] Crowe M. J. O’Connor D. Rudd D. (2007). Cold water recovery reduces anaerobic performance. Int. J. Sports Med. 28, 994–998. 10.1055/s-2007-965118 17534786

[B14] Eston R. Peters D. (1999). Effects of cold-water immersion on the symptoms of exercise-induced muscle damage. J. Sports Sci. 17, 231–238. 10.1080/026404199366136 10362390

[B15] Fielding R. A. Violan M. A. Svetkey L. Abad L. W. Manfredi T. J. Cosmas A. (2000). Effects of prior exercise on eccentric exercise-induced neutrophilia and enzyme release. Med. Sci. Sports Exerc. 32, 359–364. 10.1097/00005768-200002000-00015 10694117

[B16] Fowler N. E. Lees A. Reilly T. (1997). Changes in stature following plyometric drop-jump and pendulum exercises. Ergonomics 40, 1279–1286. 10.1080/001401397187360 9416012

[B17] Gauche E. J. Lepers R. Rabita G. Leveque J. Bishop D. Brisswalter J. (2006). Vitamin and mineral supplementation and neuromuscular recovery after a running race. Med. Sci. Sports Exerc. 38, 2110–2117. 10.1249/01.mss.0000235351.01438.5a 17146317

[B18] Hausswirth C. Louis J. Bieuzen F. Pournot H. Fournier J. Filliard J. R. (2011). Effects of whole-body cryotherapy vs. far-infrared vs. passive modalities on recovery from exercise-induced muscle damage in highly trained runners. PLoS One 6, e27749. 10.1371/journal.pone.0027749 22163272 PMC3233540

[B19] Hoffman M. D. Badowski N. Chin J. Stuempfle K. J. (2016). A randomized controlled trial of massage and pneumatic compression for ultramarathon recovery. J. Orthop. Sports Phys. Ther. 46, 320–326. 10.2519/jospt.2016.6455 27011305

[B20] Jakeman J. R. Byrne C. Eston R. G. (2010). Lower limb compression garment improves recovery from exercise-induced muscle damage in young, active females. Eur. J. Appl. Physiol. 109, 1137–1144. 10.1007/s00421-010-1464-0 20376479

[B21] Khan K. M. Thompson A. M. Blair S. N. Sallis J. F. Powell K. E. Bull F. C. (2012). Sport and exercise as contributors to the health of nations. Lancet 380, 59–64. 10.1016/S0140-6736(12)60865-4 22770457

[B22] Kim J. Kim J. Lee J. (2017). Effect of compression garments on delayed-onset muscle soreness and blood inflammatory markers after eccentric exercise: a randomized controlled trial. J. Exerc. Rehabil. 13, 541–545. 10.12965/jer.1735088.554 29114528 PMC5667600

[B23] Kraemer W. J. Bush J. A. Wickham R. B. Denegar C. R. Gomez A. L. Gotshalk L. A. (2001a). Continuous compression as an effective therapeutic intervention in treating eccentric-exercise-induced muscle soreness. J. Sport Rehabil. 10, 11–23. 10.1123/jsr.10.1.11

[B24] Kraemer W. J. Bush J. A. Wickham R. B. Denegar C. R. Gómez A. L. Gotshalk L. A. (2001b). Influence of compression therapy on symptoms following soft tissue injury from maximal eccentric exercise. J. Orthop. Sports Phys. Ther. 31, 282–290. 10.2519/jospt.2001.31.6.282 11411623

[B25] Martínez-Guardado I. Rojas-Valverde D. Gutiérrez-Vargas R. Ugalde Ramírez A. Gutiérrez-Vargas J. C. Sánchez-Ureña B. (2020). Intermittent pneumatic compression and cold-water immersion effects on physiological and perceptual recovery during multi-sports international championship. J. Funct. Morphol. Kinesiol. 5, 45. 10.3390/jfmk5030045 33467261 PMC7739238

[B26] Mattacola C. G. Perrin D. H. Gansneder B. M. Allen J. D. Mickey C. A. (1997). A comparison of visual analog and graphic rating scales for assessing pain following delayed onset muscle soreness. J. Sport Rehabil. 6, 38–46. 10.1123/jsr.6.1.38

[B27] Mizuno S. Arai M. Todoko F. Yamada E. Goto K. (2017). Wearing compression tights on the thigh during prolonged running attenuated exercise-induced increase in muscle damage marker in blood. Front. Physiol. 8, 834. 10.3389/fphys.2017.00834 29123488 PMC5662647

[B28] Murray A. Cardinale M. (2015). Cold applications for recovery in adolescent athletes: a systematic review and meta-analysis. Ext. Physiol. Med. 4, 17–15. 10.1186/s13728-015-0035-8 PMC4603811 26464795

[B29] O’Riordan S. F. McGregor R. Halson S. L. Bishop D. J. Broatch J. R. (2023). Sports compression garments improve resting markers of venous return and muscle blood flow in male basketball players. J. Sport Health Sci. 12, 513–522. 10.1016/j.jshs.2021.07.010 34314879 PMC10362518

[B30] Pournot H. Bieuzen F. Louis J. Fillard J. R. Barbiche E. Hausswirth C. (2011). Time-course of changes in inflammatory response after whole-body cryotherapy multi exposures following severe exercise. PLoS One 6, e22748. 10.1371/journal.pone.0022748 21829501 PMC3145670

[B31] Pruscino C. L. Halson S. Hargreaves M. (2013). Effects of compression garments on recovery following intermittent exercise. Eur. J. Appl. Physiol. 113, 1585–1596. 10.1007/s00421-012-2576-5 23314683

[B32] Ridard J. Rozand V. Millet G. Y. Lapole T. (2022). On-field low-frequency fatigue measurement after repeated drop jumps. Front. Physiol. 13, 1039616. 10.3389/fphys.2022.1039616 36439261 PMC9681803

[B33] Rigoard P. Ounajim A. Goudman L. Louis P. Y. Slaoui Y. Roulaud M. (2021). A novel multi-dimensional clinical response index dedicated to improving global assessment of pain in patients with persistent spinal pain syndrome after spinal surgery, based on a real-life prospective multicentric study (PREDIBACK) and machine learning techniques. J. Clin. Med. 10, 4910. 10.3390/jcm10214910 34768428 PMC8585086

[B34] Rose C. Edwards K. M. Siegler J. Graham K. Caillaud C. (2017). Whole-body cryotherapy as a recovery technique after exercise: a review of the literature. Int. J. Sports Med. 38, 1049–1060. 10.1055/s-0043-114861 29161748

[B35] Ruggiero L. Bruce C. D. Cotton P. D. Dix G. U. McNeil C. J. (2019). Prolonged low-frequency force depression is underestimated when assessed with doublets compared with tetani in the dorsiflexors. J. Appl. Physiology 126 (5), 1352–1359. 10.1152/japplphysiol.00840.2018 PMC6589818 30870083

[B36] Sarin S. Scurr J. H. Smith P. C. (1992). Mechanism of action of external compression on venous function. Br. J. Surg. 79, 499–502. 10.1002/bjs.1800790608 1611437

[B37] Schaefer E. Peil H. Ambrosetti L. Petrini O. (2003). Oedema protective properties of the red vine leaf extract AS 195 (Folia vitis viniferae) in the treatment of chronic venous insufficiency. A 6-week observational clinical trial. Arzneimittelforschung 53, 243–246. 10.1055/s-0031-1297103 12785119

[B38] Silva A. Narciso F. V. Rosa J. P. Rodrigues D. F. da Silva Cruz A. Â. Tufik S. (2019). Gender differences in sleep patterns and sleep complaints of elite athletes. Sleep. Sci. 12, 242–248. 10.5935/1984-0063.20190084 32318244 PMC7159080

[B39] Siqueira A. F. Vieira A. Bottaro M. Ferreira-Júnior J. B. Nobrega O. D. T. de Souza V. C. (2018). Multiple cold-water immersions attenuate muscle damage but not alter systemic inflammation and muscle function recovery: a parallel randomized controlled trial. Sci. Rep. 8 (1), 10961. 10.1038/s41598-018-28942-5 30026562 PMC6053395

[B40] Skurvydas A. Dudoniene V. Kalvėnas A. Zuoza A. (2002). Skeletal muscle fatigue in long‐distance runners, sprinters, and untrained men after repeated drop jumps performed at maximal intensity. Scand. J. Med. Sci. Sports 12, 34–39. 10.1034/j.1600-0838.2002.120107.x 11985764

[B41] Szabo D. A. Neagu N. Teodorescu S. Predescu C. Sopa I. S. Panait L. (2022). TECAR therapy associated with high-intensity laser therapy (Hilt) and manual therapy in the treatment of muscle disorders: a literature review on the theorised effects supporting their use. J. Clin. Med. 11 (20), 6149. 10.3390/jcm11206149 36294470 PMC9604865

[B42] Szabo Y. Z. Slavish D. C. (2021). Measuring salivary markers of inflammation in health research: a review of methodological considerations and best practices. Psychoneuroendocrinology 124, 105069. 10.1016/j.psyneuen.2020.105069 33316694 PMC8412951

[B43] Vieira A. Bottaro M. Ferreira-Junior J. B. Vieira C. Cleto V. A. Cadore E. L. (2015). Does whole-body cryotherapy improve vertical jump recovery following a high-intensity exercise bout? Open Access J. Sports Med. 6, 49–54. 10.2147/OAJSM.S70263 25750548 PMC4348140

[B44] White G. E. Wells G. D. (2013). Cold-water immersion and other forms of cryotherapy: physiological changes potentially affecting recovery from high-intensity exercise. Ext. Physiol. Med. 2, 26–11. 10.1186/2046-7648-2-26 PMC3766664 24004719

[B45] Wolska B. Domagała Ł. Kisilewicz A. Hassanlouei H. Makar P. Kawczyński A. (2023). Multiple cryosauna sessions for post-exercise recovery of delayed onset muscle soreness (DOMS): a randomized control trial. Front. Physiol. 14, 1253140. 10.3389/fphys.2023.1253140 37772056 PMC10523143

